# Targeting metastasis in paediatric bone sarcomas

**DOI:** 10.1186/s12943-025-02365-z

**Published:** 2025-05-29

**Authors:** Emma C. Bull, Archana Singh, Amy M. Harden, Kirsty Soanes, Hala Habash, Lisa Toracchio, Marianna Carrabotta, Christina Schreck, Karan M. Shah, Paulina Velasco Riestra, Margaux Chantoiseau, Maria Eugénia Marques Da Costa, Gaël Moquin-Beaudry, Pan Pantziarka, Edidiong Akanimo Essiet, Craig Gerrand, Alison Gartland, Linda Bojmar, Anna Fahlgren, Antonin Marchais, Evgenia Papakonstantinou, Eleni M. Tomazou, Didier Surdez, Dominique Heymann, Florencia Cidre-Aranaz, Olivia Fromigue, Darren W. Sexton, Nikolas Herold, Thomas G. P. Grünewald, Katia Scotlandi, Michaela Nathrath, Darrell Green

**Affiliations:** 1 Norwich Medical School, University of East Anglia, Norwich Research Park, Norwich, UK; 2 Amity Institute of Biotechnology, Amity Institute of Integrative Sciences and Health, Amity University Haryana, Gurugram, India; 3 Childhood Cancer Research Unit, Department of Women’s and Children’s Health, Karolinska Institutet, Stockholm, Sweden; 4 Laboratory of Experimental Oncology, IRCCS Istituto Ortopedico Rizzoli, Bologna, Italy; 5 Children’s Cancer Research Center, Klinikum Rechts Der Isar, Technical University of Munich, Munich, Germany; 6 School of Medicine and Population Health, The University of Sheffield, Sheffield, UK; 7 Biomedical and Clinical Sciences, Division of Surgery, Orthopaedics and Oncology, Linköping University, Linköping, Sweden; 8 Gustave Roussy Institute, Villejuif, France; 9 Department of Pediatric and Adolescent Oncology, Gustave Roussy Cancer Campus, Villejuif, France; 10 Anticancer Fund, Meise, Belgium; 11 The George Pantziarka TP53 Trust, London, UK; 12 Orthopaedic Oncology, Royal National Orthopaedic Hospital, Stanmore, UK; 13 Biomedical and Clinical Sciences, Division of Cell and Neurobiology, Linköping University, Linköping, Sweden; 14 Pediatric Hematology-Oncology, Ippokratio General Hospital of Thessaloniki, Thessaloniki, Greece; 15 St. Anna Children’s Cancer Research Institute, Vienna, Austria; 16 Center for Cancer Research, Medical University of Vienna, Vienna, Austria; 17 Faculty of Medicine, Balgrist University Hospital, University of Zurich, Zurich, Switzerland; 18 UMR6286, Nantes Université, CNRS, US2B Nantes, France; 19 Institut de Cancérologie de L’Ouest, Saint-Herblain, France; 20 Hopp Children’s Cancer Center (KiTZ), Heidelberg, Germany; 21 German Cancer Research Center (DKFZ), Division of Translational Pediatric Sarcoma Research, German Cancer Consortium (DKTK), Heidelberg, Germany; 22 National Center for Tumor Diseases (NCT), NCT Heidelberg, a partnership between DKFZ and Heidelberg University Hospital, Heidelberg, Germany; 23 Inserm UMR981, Gustave Roussy Cancer Campus, Université Paris Saclay, Villejuif, France; 24 School of Pharmacy and Biomolecular Sciences, Liverpool John Moores University, Liverpool, UK; 25 Paediatric Oncology, Astrid Lindgren Children’s Hospital, Karolinska University Hospital, Stockholm, Sweden; 26 Institute of Pathology, Heidelberg University Hospital, Heidelberg, Germany; 27 Pediatric Oncology, Klinikum Kassel, Kassel, Germany

**Keywords:** Ewing sarcoma, Osteosarcoma, Bone, Sarcoma, Metastasis

## Abstract

Paediatric bone sarcomas (e.g. Ewing sarcoma, osteosarcoma) comprise significant biological and clinical heterogeneity. This extreme heterogeneity affects response to systemic therapy, facilitates inherent and acquired drug resistance and possibly underpins the origins of metastatic disease, a key component implicit in cancer related death. Across all cancers, metastatic models have offered competing accounts on when dissemination occurs, either early or late during tumorigenesis, whether metastases at different foci arise independently and directly from the primary tumour or give rise to each other, i.e. metastases-to-metastases dissemination, and whether cell exchange occurs between synchronously growing lesions. Although it is probable that all the above mechanisms can lead to metastatic disease, clinical observations indicate that distinct modes of metastasis might predominate in different cancers. Around 70% of patients with bone sarcoma experience metastasis during their disease course but the fundamental molecular and cell mechanisms underlying spread are equivocal. Newer therapies such as tyrosine kinase inhibitors have shown promise in reducing metastatic relapse in trials, nonetheless, not all patients respond and 5-year overall survival remains at ~ 50%. Better understanding of potential bone sarcoma biological subgroups, the role of the tumour immune microenvironment, factors that promote metastasis and clinical biomarkers of prognosis and drug response are required to make progress. In this review, we provide a comprehensive overview of the approaches to manage paediatric patients with metastatic Ewing sarcoma and osteosarcoma. We describe the molecular basis of the tumour immune microenvironment, cell plasticity, circulating tumour cells and the development of the pre-metastatic niche, all required for successful distant colonisation. Finally, we discuss ongoing and upcoming patient clinical trials, biomarkers and gene regulatory networks amenable to the development of anti-metastasis medicines.

## Introduction

The origin of many paediatric cancers lies in aberrant human development [1–3]. In contrast to adult cancers in which exogenous mutagens or age accumulated DNA damage drives tumour development, paediatric cancers lack the extended time frame required to accumulate the mutations required for tumorigenesis by these routes [4]. Endogenous in utero mutagenic processes are a likely source for cancer inducing mutations in paediatric cancers. The childhood tumour spectrum is mostly unique and shows a predilection for particular age groups [5] suggestive that the cell of origin in paediatric malignancies is absent in adult tissues [1]. Childhood tumours are rare and typically involve few driver events indicating that there is a limited biological window for tumour formation, alluding to the transiency of the cell of origin [1]. Adult cancers are frequently of epithelial origin and increase in prevalence with age [5]. Conversely, many paediatric tumours are derived from mesodermal and ectodermal lineages. At the molecular cell level, paediatric cancers are generally characterised by terminal differentiation failure [1, 6–10], epigenetic changes [11], gene rearrangements [3, 12], low mutational burden [13, 14] and low T cell activity [14]. When paediatric and adult cancers are histologically similar, there are usually distinctive discriminating features. For example, childhood osteosarcomas harbour *TP53* and *RB1* driver mutations whereas adult osteosarcomas are usually secondary to *SQSTM1* positive Paget’s disease of bone [15–19] or radiation exposure [20, 21]).

An important observation in humans indicating a developmental origin of paediatric cancer is in childhood acute lymphoblastic leukaemia, which evolves in two discrete steps [22]. First, in utero initiation where fusion gene formation (*ETV6*::*RUNX1*) generates a pre-leukemic clone [22]. Second, in a small fraction of these cases and sometimes with a protracted latency of 1–15 years [23], postnatal acquisition of secondary genetic changes drives conversion to overt leukaemia [22]. The bone sarcoma cell of origin is debated [24] but experimental studies in different models suggest similar in utero mechanisms. In Ewing sarcoma, *FET*::*ETS* gene fusions, most commonly *EWSR1*::*FLI1* [25], are generated either by balanced chromosomal translocations or loop like rearrangements [26] termed chromoplexy [27]. The in-frame encoded fusion oncoproteins create de novo enhancers at repetitive GGAA DNA microsatellites [28]. These neoenhancers appear to contribute to tumorigenesis and eventually tumour progression and possibly underlie germline variation [28–36]. A study in Zebrafish reported that conditional expression of the *EWSR1*::*FLI1* transgene in a trunk neural crest cell may cause transcriptional hijacking and mesoderm lineage reprogramming, which might underlie the formation of neoplasms reminiscent of human Ewing sarcoma [37]. A human case report showed the *EWSR1*::*FLI1* mutation arising in a mesenchymal stem cell [38]. Mimicking Ewing sarcoma in mice has been challenging [39], however, a recent mouse model showed that while *EWSR1*::*FLI1* may be sufficient for tumorigenesis, subsequent YAP1 activation induced by IGF1 signalling may be required for the activation of TEAD driven transcription and metastatic progression [10]. Thus, multiple cells of origin may be possible for Ewing sarcoma.

The cell of origin topography is more obscure in osteosarcoma because the multitude of aberrations present in osteosarcoma genomes complicates most discovery studies [40–42], however, *TP53* or *RB1* loss-of-function [43, 44] or mutant gain-of-function [45, 46] in an osteoblastic like progenitor are accepted. In utero imprinting defects at the chromosome 14q32 locus have been reported, affecting *DLK1*, *RTL1*, *DIO3*, *MEG3*, *MEG8* and *DIO3OS* gene expression plus the expression of over 40 microRNAs (miRNAs), some involved in *MYC* regulation, bone differentiation and pluripotent stem cell reprogramming and speculated to predispose affected individuals to osteosarcoma development [47]. In untreated clinical samples, driver mutations likewise to *TP53* and *RB1* were identified in Mendelian cancer driver genes *BRCA2*, *BAP1*, *RET*, *MUTYH*, *ATM*, *PTEN*, *WRN* and *RECQL4* and cancer susceptibility genes *ATRX*, *FANCA*, *NUMA1* and *MDC1* [48]. Any one of these 14 drivers is proposed to be responsible for chromosomal instability and osteosarcoma development [48]. Single cell tracking in an induced murine osteosarcoma model showed osteosarcoma cells present initial polyclonal dynamics followed by local clonal dominance with metastases arising clonally or polyclonally but with a different cellular origin than the dominant clones in the primary tumour, suggestive of a neutral evolution model [49]. In human osteosarcoma tumours, chromothripsis was found to be an ongoing mutational process, which mediated punctuated evolution of the disease [50].

Despite that the genetic driver mechanisms are reasonably understood for Ewing sarcoma and osteosarcoma, trials investigating targeted therapies and immunotherapies have not progressed to standard of care [51, 52]. Morbidity is still high and survival rates remain low, especially in the metastatic setting [51], where pulmonary metastasis causes death by respiratory failure or infection [53]. Disseminated and refractory disease remains the leading oncology challenge. Translational research to better understand systemic disease and to design new clinical opportunities is likewise challenging because there is difficulty in obtaining metastatic samples due to reduced surgical intervention at this clinical stage [51]. Drug resistant and inoperable metastases therefore remain the leading cause of bone sarcoma death [54]. Intervention of this specific disease component, which is thought to be independent and biologically highly distinct from tumorigenesis, might have a significant impact on outcomes.

**Fig. 1:**
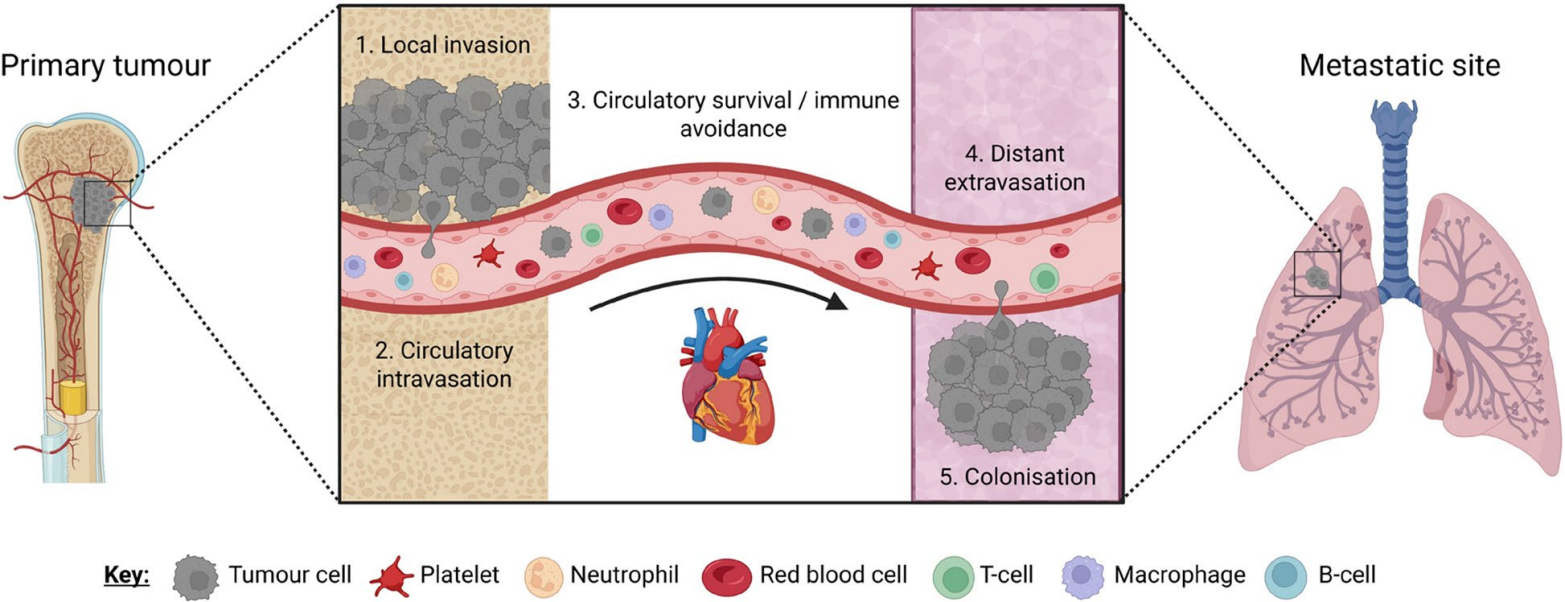
Metastasis is the defining and fatal feature of cancer. Some primary tumour cells, through multiple mechanisms, invade the local vasculature and spread around the body using the blood circulatory system. In some cases, this could also be the lymphatic system. These so-called circulating tumour cells survive circulatory cytotoxicity, avoid immune detection and invade distant sites to propagate secondary tumours (metastases). Metastases are often drug resistant and generate at inoperable sites meaning their growth and further spread involves the dysfunction of multiple interconnected systems within the body, ultimately leading to patient death. Clinical observations indicate that some primary tumours show a proclivity towards specific distant sites. For example, melanomas tend to spread to the brain. Breast cancers spread to the bones, etc. As depicted, bone sarcomas tend to spread to the lungs

Contemporary understanding of the metastatic evolution of cancer cells (Fig. 1) is described by linear Darwinian evolution [55], where tumour cells temporally acquire selected and heritable changes, consequently, primary tumours and metastases are genetically closely related, or the parallel progression model, where dissemination occurs in the early stages of the disease and metastases and the primary tumour evolve independently resulting in genetic disparity [56]. Comparative genomics studies performed in different cancers describe a diversity of possible progression trajectories for metastatic disease [56]. It could also be possible that a metastatic clone already exists at the very beginning of tumorigenesis and needs time to expand. Rather than cumulative mutational burden over time, all mutations driving metastasis are present ab initio.

While some driver mutations impact the expression and/or function of a single protein, others may influence the expression of multiple genomically adjacent and/or more distant genes [57–59] exerting global effects. For example, through mutations in epigenetic modifiers, genes modulating splicing or through effects on downstream transcription factors [60–65]. Copy number variants, amplifications, structural variants and gene fusions, all frequent and typical sarcoma features, usually have a more extensive transcriptional impact than point mutations [58, 60, 66].

Cancer cell clonal selection is not only contingent on genetic mutations [67]. Any heritable alteration whether genetic or not may be subject to selective pressures [68]. Epimutations, described as aberrant epigenetic patterns that drive specific oncogenic phenotypes, [69] including hypermethylation affecting tumour suppressor and cell cycle genes [70, 71] and changes to three dimensional chromatin topology [72, 73] are all implicated in cancer evolution [67]. The surrounding cell environment and metabolic adaptation might also influence phenotypic plasticity where malignant cells can access states or phenotypes that were not directly inherited from an ancestor [67].

In this review, we capitalise on a renaissance in the paediatric bone sarcoma space, with two international panels of scientists, clinicians and patient and parent advocates forming the EURO EWING Consortium (EEC) and the Fight Osteosarcoma Through European Research (FOSTER) consortium to address the problem that there have been no new bone sarcoma medicines since the introduction of chemotherapy in the 1970 s [74–77]. Clinical guidelines for diagnosis, treatment and follow up were updated in 2021 [78] and 2024 [79]. Research guidelines for sample collection and structured analysis were established in 2024 [51, 80]. Here we describe how metastatic bone sarcoma is best managed in the clinic, we comprehensively outline our most recent understanding of the molecular and cellular mechanisms that underpin bone sarcoma metastasis, pose future research questions and discuss clinically relevant opportunities [51, 78].

### Chemotherapy combined with surgery

As almost all paediatric patients with bone sarcoma developed metastases in the short term with local therapy alone, chemotherapy was added to the clinical protocol in the 1970 s [74–77]. Local radiotherapy was included for some Ewing sarcoma cases. The combination of systemic and local therapy led to significant improvements in outcome. Actuarial 10-year survival rates increased from 10 to 40% and even higher in localised disease cases [81, 82]. The success of preventing metastatic relapse in both Ewing sarcoma and osteosarcoma using systemic combination chemotherapy emphasises that bone sarcomas should be considered a systemic disease with radiologically undetectable micrometastases already present at diagnosis. Overt metastatic disease detected at presentation confers a poor outcome with < 20% long term survival [81]. It is unknown whether the inferior outcomes in these metastatic cases as compared to localised disease correspond to differences in tumour biology but it seems likely that systemic treatment efficacy is limited by pharmacokinetic barriers of macroscopic tumour masses [83]. This consideration implies that similarly stringent surgical and/or radiotherapeutic criteria should be applied where feasible to achieve local control even at metastatic sites.

Almost one-third of patients with Ewing sarcoma and osteosarcoma present with detectable metastases [78]. Secondary lesions are typically pulmonary, less commonly bone/bone marrow [84, 85]. “Skip” metastases, sometimes observed in osteosarcoma and less so in Ewing sarcoma, occur in the same and/or adjacent bone as the primary tumour and represent local regional spread [86]. Skip lesions should be resected at the same time as the primary tumour. For curative intent in the metastatic disease setting, all lesions should be removed completely by surgery where feasible. Due to osteosarcoma radiation insensitivity, high dose radiotherapy of selected metastases using techniques such as proton or heavy ion therapy should only be considered if surgical treatment is not possible [87]. For Ewing sarcoma, complete surgical excision including all metastases is the best modality of local control but there are specific clinical situations where radiotherapy addition might be useful [88]. Chemotherapy protocols intended at treating metastases do not differ from those used for localised disease. Chemotherapy is administered according to national guidelines [79, 89]. In paediatric osteosarcoma the chemotherapeutic backbone comprises combined high dose methotrexate, doxorubicin and cisplatin [79]. For Ewing sarcoma, chemotherapy comprises vincristine, doxorubicin and cyclophosphamide alternated with ifosfamide and etoposide [90].

Bone sarcoma relapses mainly occur 1–2 years after definitive treatment. Relapse is rarely observed at > 10 years. Local therapy objectives for metastases are the same as for the primary tumour: complete surgical removal with wide margins at least half the size of the pulmonary node [91, 92]. Ewing sarcomas can be with a narrow resection complemented by radiotherapy [79]. The goal of metastasectomy is limited resection with maximum preservation of normal lung tissue. Nodules can be detected intraoperatively by palpation. Deeper, smaller and softer nodules can be preoperatively marked with wires, coins or paint plus intraoperative ultrasound to locate the lesion. Long term survival can be achieved through surgical resection of subsequent uni- or oligometastatic relapses especially in osteosarcoma [93, 94] where 5-year survival increases to 60% and 20-year survival to 30% with repeated metastasectomies [95–97]. Radiotherapy used as definitive therapy is an effective procedure for local Ewing sarcoma control but less effective than surgery [79] and mainly used for inoperable lesions [98]. Video assisted thoracoscopic surgery (VATS) is favoured for its minimally invasive approach, shorter recovery periods and oncologic outcomes comparable to traditional thoracotomy [79].

The value of further adjuvant systemic therapy for relapsed osteosarcoma is debated. The Cooperative Osteosarcoma Study Group suggest that there is only a limited increase in cure rate with additional cytotoxic chemotherapy [99]. Second-line ifosfamide/etoposide and carboplatin/etoposide are the commonest agents used if so. Results from closed trials investigating adjuvant mifamurtide (SARCOME13, #NCT03643133) and denosumab (#2021–002366-41) in metastatic osteosarcoma are expected soon. In relapsed Ewing sarcoma, several multiagent therapies have demonstrated clinical activity. High dose ifosfamide was shown to be more effective in prolonging survival than other commonly used drugs including topotecan/cyclophosphamide, irinotecan/temozolomide or gemcitabine/docetaxel [100]. Three-year additional maintenance therapy with metformin is under evaluation for high-risk bone sarcomas (Metform-Bone, #NCT04758000). Six-month additional maintenance treatment using vinorelbine/cyclophosphamide in high-risk Ewing sarcoma is under evaluation (iEuroEwing, #2019–004153-93). Tyrosine kinase inhibitors (TKIs) have shown promise in Ewing sarcoma and osteosarcoma [101]. High dose ifosfamide combined with the TKI lenvatinib is under evaluation in Ewing sarcoma (rEECur, #2014–000259-99). Regorafenib (INTER-EWING-1, #2021–005061-41) and cabozantinib (#NCT05691478) combined with chemotherapy are under evaluation in Ewing sarcoma and osteosarcoma, respectively [102].

### Post-surgical metastatic relapse

**Fig. 2:**
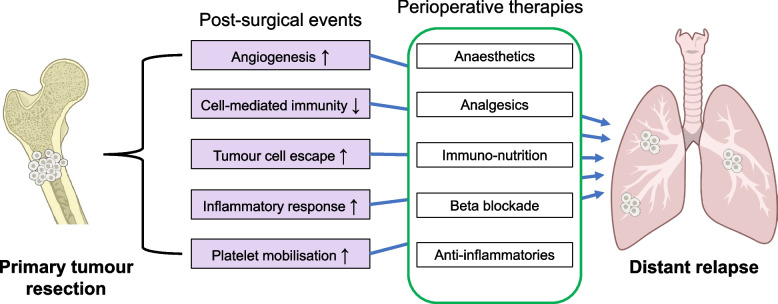
Post-surgical metastatic relapse following primary tumour resection and potential mechanisms of action

Surgical removal of tumours is required for cure but patients with all cancer types including bone paradoxically experience a high relapse rate following surgery [103]. One suggested mechanism for post-surgical associated metastatic relapse is that major surgery trauma inherently induces growth factor and cytokine secretion supporting tissue repair and angiogenesis followed by immunosuppression [104–106]. Together these processes create an environment conducive to micrometastatic growth. It is possible that short term interventions reducing these pro-metastatic physiological conditions might reduce disease relapse and increase survival [107–109]. A number of such interventions termed perioperative therapies are under evaluation [103, 110] (Fig. 2).

There is in vitro evidence that at sub-clinical concentrations the anaesthetic propofol inhibits the invasive abilities of human cancer cells including the HOS osteosarcoma cell line and decreases pulmonary deposits in vivo [111]. Retrospective data from 100 patients with bone sarcoma showed that regional anaesthesia was associated with increased metastasis free survival [112]. Patients with osteosarcoma undergoing limb salvage surgery using propofol anaesthesia rather than desflurane anaesthesia had improved overall survival and a lower risk of post-operative recurrence and metastasis [113]. These findings require prospective validation. Different anaesthetic agents impact post-surgical immune function in patients with osteosarcoma [114]. A recent bone sarcoma study revealed a significant decrease in natural killer (NK) cells plus subsequent increase in the pro-inflammatory cytokine IL6 following surgery [115]. Immune activation via post-operative infection enhances bone sarcoma survival [116]. Immune function is associated with osteosarcoma relapse risk, but not tumour growth, as demonstrated by immune reconstitution in immunodeficient mice [117].

## Clinically relevant biomarkers

An important issue for patients newly diagnosed with bone sarcomas is that the clinical outcome is highly variable. Precise prediction of disease progression through biomarkers is not possible despite much research in this area [118, 119]. Given some of the mechanisms of action associated with early post-surgical relapse, there are a number of serum/plasma biomarkers of interest with some evidence of biological relevance in bone sarcoma (Table 1).

**Table 1:** Clinically relevant bone sarcoma serum/plasma biomarkers

Serum/plasma biomarker	Relevance
Vascular endothelial growth factor (VEGF)	Elevated pre-treatment serum VEGF associated with a worse osteosarcoma prognosis. Associated with distant relapse, not local recurrence [120, 121]
C-reactive protein (CRP)	Elevated pre-treatment serum CRP associated with reduced osteosarcoma overall survival [122, 123]
Alkaline phosphatase (ALP)	Elevated post-operative or post-chemotherapy serum ALP associated with reduced osteosarcoma event free or overall survival [124]
Lactate dehydrogenase (LDH)	Elevated serum LDH associated with reduced Ewing sarcoma and osteosarcoma event free survival [125]
Neutrophil/lymphocyte ratio (NLR)	High pre-treatment neutrophil to lymphocyte ratio associated with reduced osteosarcoma overall survival [123, 126]
Platelet/lymphocyte ratio (PLR)	High pre-treatment platelet to lymphocyte ratio associated with reduced osteosarcoma overall survival [127]
Ceruloplasmin	High plasma levels associated with a metastatic Ewing sarcoma disease profile [128]

In multiple cancer types the platelet: lymphocyte ratio (PLR) is a prognostic marker. Increased PLR or platelet counts are associated with an increased risk of metastatic spread and reduced survival [129–131]. Pre-operative PLR might relate to osteosarcoma outcomes [127]. A prognostic index to predict 5-year overall and metastasis free survival was developed, which included pre-treatment platelet and neutrophil counts as part of a validated clinical model [132]. In Ewing sarcoma, patient derived plasma proteomic profiling identified ceruloplasmin as a prognostic marker for patients with metastatic disease [128] (Table 1). TCF7L1 is also prognostically relevant in metastatic Ewing sarcoma [133].

### Tumour heterogeneity

Nuclear medicine techniques show that the minimum size of a detectable lesion is ~ 1.5 mm^3^ [134], however, this small tumour will already comprise around 150 million cancer cells with divergent temporal and spatial phenotypes unique to the individual patient including drug resistance and metastatic propensity. The role of intratumoral heterogeneity in disease spread has mostly been described in carcinomas [135–137]. Housing different (sub)clones not only provides the foundations for different evolutionary trajectories but the tumour as a whole becomes more resilient and adaptive to intrinsic and extrinsic stressors including chemotherapy. The more diverse a tumour the increased likelihood for overcoming local challenges, for example, acidosis, hypoxia, and chemotherapy, and then initiating metastasis. Intratumour genetic heterogeneity might also produce immune-escape proficient clones [138]. Conversely, increased genetic diversity may lead to increased immunogenicity, reducing tumour ability to evade immune detection [139]. Heterogeneity can confer both tumour advantages and disadvantages, indicating the obligation for a ‘balance’ required for the tumour to progress and disseminate.

In osteosarcoma, the biological/clinical impact of intratumoral heterogeneity on metastasis remains unclear despite several studies aiming to elucidate its possible, and potentially finite, evolutionary trajectories [41]. There is evidence for clonal selection during the epithelial-to-mesenchymal (EMT) and mesenchymal-to-epithelial (MET) processes prior to metastasis, indicated by the presence of a subset of clones in metastatic lesions when compared to the primary [140, 141]. WNT, a family of signalling pathways known to perform roles in cell fate determination, cell migration and tissue development [142] combined with NOTCH, a cell-to-cell communication pathway central to various developmental processes [143] as well as oxidative phosphorylation (OXPHOS) underlie these EMT/MET transitions [140, 141]. Osteoblastic osteosarcomas may have greater metastatic potential than other histotypes such as chondroblastic [141] suggesting phenotypic heterogeneity. While these early observations are important, their generalisability is limited. One study focused on lymph node metastases, which are uncommon in osteosarcoma. Another used a small number of unpaired samples for primary and metastatic groups, which precluded using matched comparisons.

In contrast to other cancers including osteosarcoma where there are molecularly defined disease subtypes [48, 144–146], Ewing sarcoma demonstrates a greater emphasis on phenotypic heterogeneity because of its relative genetic inertness. One of the principal heterogeneity sources derive from the expression level of the EWSR1::FLI1 chimeric oncoprotein. Tumour cells are thought to reside in a dynamic and metastable state fluctuating between ‘high’ or ‘low’ EWSR1::FLI1 expression [32, 147–149]. These expression states have phenotypical implications because tumour cells residing in an EWSR1::FLI1^high^ state depict strong proliferative features whereas cells in an EWSR1::FLI1^low^ state show a predominantly migratory/metastatic phenotype [147, 148]. Ewing sarcoma genetic heterogeneity might also arise from additional mutations in *TP53* (~ 7%) and *STAG2* (~ 17%), copy number variants [150, 151] and/or chromosomal gains and losses [152–159]. *STAG2* mutated Ewing sarcomas are associated with increased metastasis and poorer clinical outcomes [152–155]. These mutated cells combine high proliferation similar to EWSR1::FLI1^high^ cells with enhanced invasiveness resembling EWSR1::FLI1^low^ cells [155, 156]. Mechanistically, mutations in this cohesin member do not alter EWSR1::FLI1 levels but reduce its cis-mediated activity by reshaping the dynamics of chromatin loop formation [155]. There is DNA hypomethylation at enhancer regions regulated by EWSR1::FLI1 [32] but differential DNA methylation between tumours suggest a continuous disease spectrum reflecting EWSR1::FLI1 regulatory signature strength, a continuum between mesenchymal and stem cell signatures potentially emulating the regulatory cell state from which the tumour originally derived [32]. DNA methylation levels/differences are more pronounced in patients with metastatic disease when compared to local disease [32]. This discovery supports the growing consensus that tumour heterogeneity is often greater in more aggressive cancers [160, 161].

### Mineral bone environment

The primary tissue site is potentially important for generating metastatic cells, i.e. malignant bone sarcoma cells are required to be in bone tissue to generate metastatic cells. A prostate cancer study showed that ectopic tissue sites fail to produce metastatic cells [162]. Where Ewing sarcoma cells are purportedly derived from one of the neural crest [163] trajectories [37] and/or the mesenchyme [38], osteosarcoma cells are committed to the osteoblastic lineage ranging from a mesenchymal stem cell to pre-osteoblast progenitor to mature differentiated osteoblast. This differentiation spectrum may contribute to the diversity of conventional osteosarcoma histotypes including osteoblastic, chondroblastic, fibroblastic or mixed [164]. Malignant transformation does not divert the cell from its osteoblastic roadmap. Osteosarcoma lesions produce an organic collagenous extracellular matrix (osteoid) that is more or less organised and mineralised. The latter is the main feature not shared by any other connective tissue. Around 60% of pulmonary nodules in patients with metastatic osteosarcoma exhibit calcification as evaluated by computed tomography (CT) scans [165–167] indicating that disseminated osteosarcoma cells still maintain a mineralisation capacity even outside of bone tissue. This inherent biology is a valuable asset for the follow up monitoring of patients by conventional chest CT imaging as well as bimanual palpation during thoracotomy.

Calcium and phosphate salts are the principal bone forming minerals. These salts associate into hydroxyapatite (Ca_10_(PO_4_)_6_OH_2_) crystals. Selenium doped calcium phosphate (Se-CaP) biominerals used as a drug carrier supported multidrug resistance (MDR) reversal in doxorubicin resistant MG63 cells by inducing the downregulation of MDR associated ATP-binding cassette transporters (ABCB1 and ABCC1) [168]. Selenium oxide has been shown to reduce tumour growth but not to prevent tumour incidence when administrated in the drinking water of nude mice implanted with a KOS cell xenograft [169].

Important ions, for example, magnesium, zinc, copper, potassium, fluoride, sodium, manganese, silver, iron and boron are stored in bone tissue [170]. Bone remodelling provides the skeleton and the rest of the body with a usable reservoir [171]. Despite a trace element, the skeleton accounts for ~ 30% of the body’s overall zinc content [172]. Zinc plays key roles in enhancing bone metabolism by favouring osteoblastogenesis and preventing resorption. Adequate zinc levels are required for the expression of osteoblastic and in some cases metastasis markers, for example, *RUNX2*, *ALP*, *OC* and *COL1 A1* [54] and suppression of the osteoclast differentiation markers bone tartrate resistant acid phosphatase (TRAP) and cathepsin K.

Inverse copper and zinc fluctuations have been reported in the serum of patients with primary and metastatic osteosarcoma. Both local and systemic disease presents elevated serum copper levels whereas surgically treated patients have nearly normal levels [173]. Patients with primary osteosarcoma have elevated serum zinc, those with metastases have reduced zinc and surgically treated patients have nearly normal serum zinc [173]. The serum copper: zinc ratio in metastatic osteosarcoma is higher than primary osteosarcoma, therefore, a possible tool for discriminating patient stage [173].

Several studies have proposed the incorporation of zinc into hydroxyapatite to produce biomaterials (ZnHA) that stimulate and accelerate bone healing [174]. Other biomaterials such as gallium doped bioactive glasses and Mg/Zn or Cu/Zn scaffolds can reduce osteosarcoma local recurrence and accelerate tissue repair after surgery [175, 176]. Nanoparticles potentially improve the efficacy of chemotherapeutic or targeted drugs. Doxorubicin loaded into iron oxide nanoparticles exhibited enhanced in vitro cytotoxic effects on MG63 osteosarcoma cells [177]. Nanoparticle conjugation triggers drug internalisation by micropinocytosis and subsequent accumulation in the perinuclear region; therefore, easier access to DNA, though it is noted that MG63 cells produce a ‘weaker’ extracellular matrix that is not entirely analogous to bone.

Bisphosphonates, for example, zoledronate, alendronate, risedronate and pamidronate are a drug class structurally similar to pyrophosphate but with a central carbon that can have up to two substituents, R^1^ and R^2^, instead of an oxygen atom [178, 179]. Because a bisphosphonate mimics the structure of pyrophosphate, it can inhibit the activation of enzymes that utilise pyrophosphate. Bisphosphonate based drug specificity comes from the two phosphonate groups that work together to coordinate calcium ions, as bisphosphonate molecules preferentially bind to calcium. The largest store of calcium in the human body is in bones, so bisphosphonates accumulate to a high concentration in bones. Bisphosphonates when attached to bone tissue are released by osteoclasts where they disrupt intracellular enzymatic functions required for bone resorption [180]. This bone enhancement effect was proposed as a therapeutic approach to block bone resorption and bone tumour induced osteolysis [180]. Preclinically, bisphosphonates significantly reduced Ewing sarcoma and osteosarcoma growth and pulmonary metastasis but in combination with chemotherapy and surgery in randomised phase III studies there was no improvement in clinical outcome [181, 182]. Zoledronate clinical inefficiency in the bone sarcoma context is thought to be explained by the biological impact on macrophage differentiation and recruitment and negatively altering CD8 + killer T cell tumour infiltration [183]. Reduced CD8 + levels were associated with metastatic disease and reduced overall survival [183]. Despite the lack of benefit as a therapeutic agent, bisphosphonates remain an excellent drug delivery platform to bone because of their considerable affinity for the mineralised extracellular matrix, which opens up new opportunities for their future use [180].

### Tumour microenvironment

**Fig. 3:**
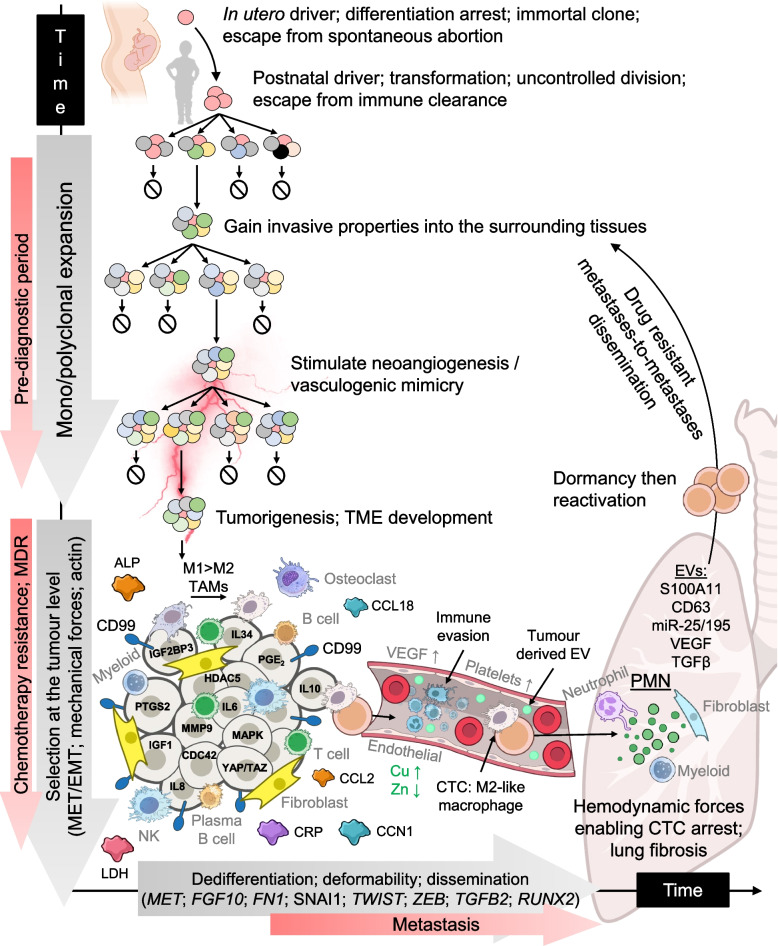
Panoramic overview of bone sarcoma multi-step metastasis and targets for anti-metastasis medicines. At the top of the figure, the schematic portrays the predicted scenario where the bone sarcoma cell of origin arises during, and arrests in, development caused by rare mutations in specific cell populations during restricted developmental windows. This precursor cell may require secondary activation, for example, hormone onset at adolescence, before mono- or polyclonal expansion and invasion into local tissues. The bottom left of the figure depicts the established primary bone tumour with the multitude of other interacting cells, molecules and genes associated with metastatic propensity, and all potential targets for new therapies. The figure shows CTC escape and into the local blood vasculature where there are reported ion differences between patients with and without metastatic disease as well as increased platelets. The bottom right of the figure displays the PMN, typically the lungs, where new cell types, EVs and genes have been associated with the arrest and propagation of CTCs. Finally, these disseminated cells form secondary tumours, that may themselves shed CTCs enabling metastases-to-metastases dissemination

The tumour microenvironment (TME) is a complex cell and molecule ecosystem surrounding and interacting with a tumour (Fig. 3). TME composition is variable between patients even with the same cancer type. Composition and function depends on several factors including inflammation, hypoxia, neoangiogenesis (NA) and vasculogenic mimicry (VM) [184] (Fig. 3). There can be a rich cell type diversity. Single-cell RNA sequencing (scRNA-seq) performed on untreated osteosarcomas revealed nine major cell types in the TME including osteosarcoma cells, myeloid 1 and 2, osteoclasts, NK/T cells, B cells, fibroblasts, endothelial cells and plasma B cells [185] (Fig. 3). Immunotherapies that target tumour-stroma interactions instead of tumours directly have shown efficacy in several sarcomas [186].

**Fig. 4:**
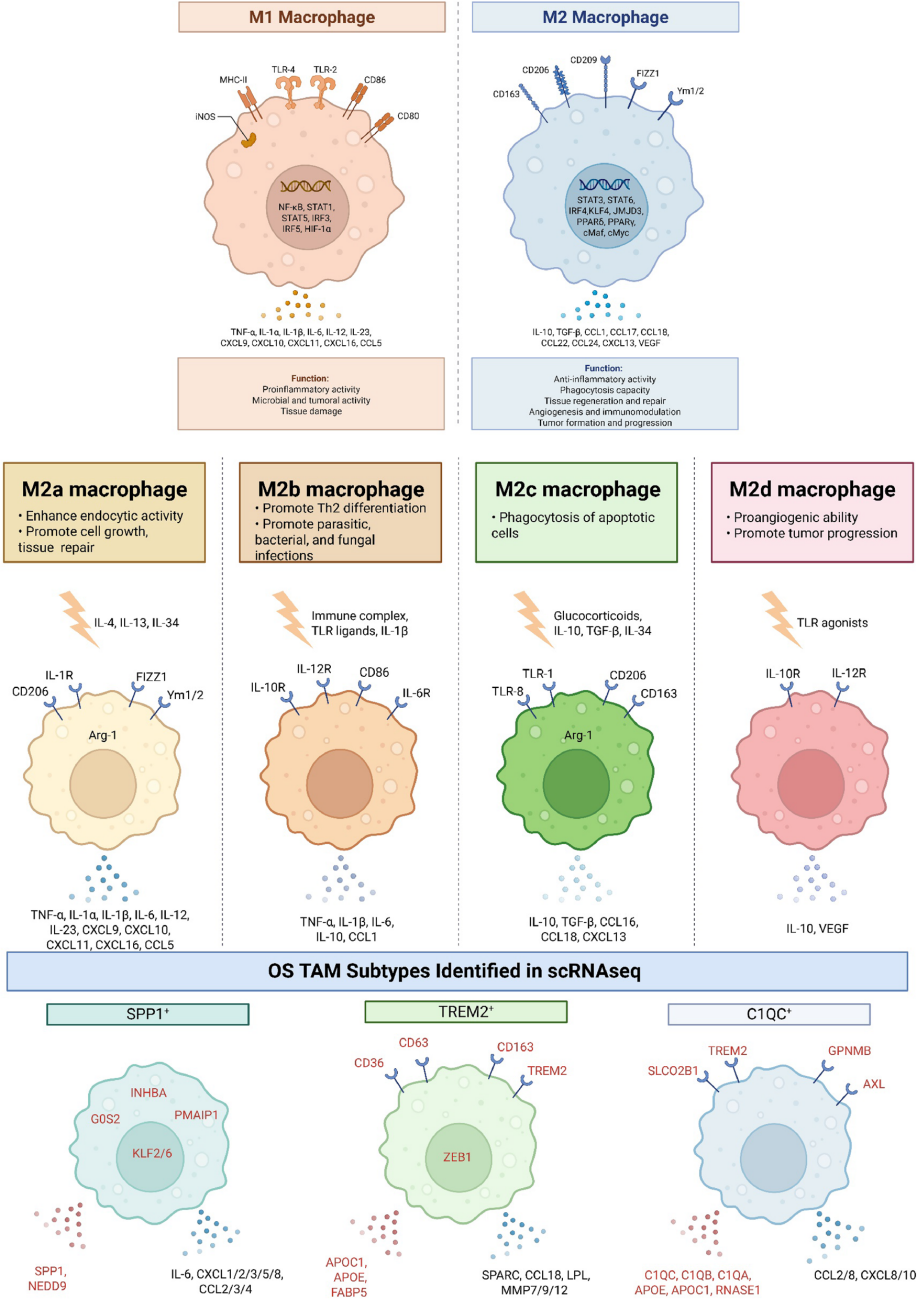
Macrophages have been classically defined as M1 and M2 with M2 further categorised into subtypes. TAM heterogeneity blurs these divisions but generally lead to the predominant pro-tumour survival features of M2 phenotypes. scRNA-seq has enabled newer TAM subtypes to be identified and in osteosarcoma, three are displayed here. Signature genes for each subtype are in red

The TME and tumour perpetually influence each other. For example, tumour derived signals confer macrophage phenotypes, which reciprocally support tumour and disease progression. Circulating monocytes, regulated by M-CSF, differentiate into naive resting macrophages, M0, when they move from the bloodstream into tissues. Activated macrophages then broadly exist as two polarised phenotypes: classically activated pro-inflammatory, M1, which produce pro-inflammatory cytokines and nitric oxide and initiate an immune response and alternatively activated anti-inflammatory, M2, which promote wound healing and repair [187] (Fig. 4).

Sarcoma TMEs are abundant with M2 like tumour associated macrophages (TAMs) [187, 188], which represent around 50% of the immune cell population [188, 189]. TAMs are extremely plastic and differentially polarise. Within M2 TAMs there is further classification. For example, CD68 + and CD163 + M2 TAMs are distinct cell types with specific functions. CD68 +/CD163 + levels have been associated with osteosarcoma clinical outcome: higher CD163 + levels were associated with better overall survival and longer metastasis free survival whereas CD68 + showed no association [183]. CD68 + M2 TAMs detected in untreated osteosarcomas were measurably similar between local and metastatic disease but M1 TAMs were significantly more abundant in non-metastatic than metastatic patients [186].

scRNA-seq has further defined TAM subtypes with SPP1 +, TREM2 + and C1QC + subtypes in osteosarcoma [190]. SPP1 + functions to promote NA and recruit immune cells [141]. TREM2 + is associated with lipid metabolism, immunosuppression and matrix remodelling [141]. C1QC + is linked to phagocytosis and tumour progression [191]. Clinically manipulating the TME through M1/M2 modulation has recently been explored in Ewing sarcoma [192]. CD99 ligation induced M2 TAM to M1 reprogramming resulting in reduced tumour growth in patient derived xenografts [192]. In osteosarcoma, tumour derived MAPK7 was shown to promote F4/80 + M2 TAM polarisation [54]. Mutant osteosarcomas lacking MAPK7 through stably expressed RNAi failed to metastasise in vivo [54].

Beyond their role in modulating inflammation in the TME, TAMs also secrete factors that actively enhance tumour growth, invasion, NA/VM and metastasis. Several TAM derived chemokines and interleukins including TGFβ, IL6, IL10, CCL2 and CCL18 are essential for metastatic bone sarcoma with most studies performed in osteosarcoma [193–198] (Fig. 4). In endocrine resistant breast cancer, TAM derived CCL2 was reported to be the causal culprit for tumour drug resistance via its activation of tumoural PI3 K/AKT/mTOR signalling [199], a pathway often implicated in bone sarcomas. In osteosarcomas under sheer stress, tumour derived IL34 increases TAM recruitment exacerbating NA/VM, tumour growth and metastasis [200] (Fig. 3). High CCL18 levels in serum and osteosarcoma tissues derived from CD68 + M2 TAMs are associated with lung metastases development [201]. FABP4 + TAMs have been reported as the predominant macrophage phenotype in osteosarcoma derived lung metastases [191]. FABP4 expression encourages fatty acid metabolism in secondary lesions [191]. The lack of available pharmaceuticals against molecular targets makes TAMs excellent cell targets for anti-metastasis approaches, for example, trabectedin [202], in bone sarcoma treatment.

### Immunometabolism

Non-immune cell metabolism that affects immune cell state and fate, termed immunometabolism, contributes to infectious disease, inflammation and cancer [203]. Cancer cell metabolic adaptation generates a TME in which immune cells lose their cancer killing capacity [141, 204, 205] despite the development of immune checkpoint therapies [206]. Immune cells in the TME also develop distinct metabolic characteristics. CD4 + helper T cells and CD8 + killer T cells conform to the Warburg effect of obtaining energy via glycolysis rather than the more efficient tricarboxylic acid cycle [207]. CD3 +/CD25 + regulatory T cells and CD45RO + memory T cells, however, continue to derive most of their energy from OXPHOS [206]. T cell metabolic dysfunction might result in a loss of immune function against tumours [208]. TME factors can drive these immunometabolic changes. For example, hypoxia, nutrient deprivation and lactate accumulation could contribute to immunometabolic associated metastasis. Bone sarcoma specific *IDH1*/*2* mutations [209, 210] causing abnormal isocitrate metabolism yielding the oncometabolite 2-hydroxyglutarate rather than α-ketoglutarate require investigation of their possible immunometabolic effects [70].

Rewiring metabolism in tumour and immune cells is clinically viable. Succinate delivery to the melanoma TME through polyethylene succinate microparticles has been shown to support M1 like TAM phenotype maintenance and subsequent CD4 + helper T cell responses [211]. Hypoxia modifies TAM plasticity leading to increased glycolysis and altered fatty acid metabolism [212]. Since TAMs rely on fatty acid oxidation (FAO) for their energy needs, targeting FAO could impair their survival/function. FAO inhibitors including etomoxir reduce TAM immunosuppressive activity and boost the efficacy of anti-tumour immune responses [212]. TAMs metabolise the abundant tumour derived lactate, which further facilitates their survival in the acidic TME. Inhibiting LDH, which converts pyruvate to lactate, can reduce TME lactate levels and promote a phenotypic shift from M2 like to M1 like [213]. Immunometabolism as a clinical research field may yield improved anti-metastatic immunotherapies.

### Tumour biomechanics

Tumour cells are surrounded by changing mechanical forces including compression, hydrostatic pressure, shear stress and tension forces (Fig. 3). These forces are dynamic during tumour cell detachment and movement through the extracellular matrix or across endothelial barriers before entering the blood circulation and/or the lymphatic system. Forces have a significant impact on the metastatic cell phenotype during invasion [214–217]. Bone sarcomas are confronted with a uniquely challenging biophysical environment given their location inside skeletal tissue that undergoes a continuously changing mechanical environment during normal bone remodelling. The TME located in bone is highly complicated with frequent interactions between tumour, stromal, immune and bone cells (Fig. 3). Mechanical forces including solid and shear stress are generated subsequent to uncontrolled cell division and the production of new extracellular matrix [218, 219]. Cell adaptation to biophysical forces involves biochemical and biomechanical signalling leading to changes in intra- and extra- cellular communication [214, 217]. Understanding how mechanical forces influence the TME and downstream communication in bone sarcoma progression might lead to new targets and therapies.

### Sheer stress: a direct response

Osteosarcoma cells can respond to shear stress through integrins, transmembrane receptors supporting cell–cell and cell-extracellular matrix adhesion. Upon ligand binding integrins can activate signal transduction pathways. Integrins directly respond to the MAPK pathway [220, 221] and IGF1 [222] in osteosarcoma under mechanical stimulation. MAPK overexpression promotes various proliferative signalling networks driving cancer cell division through cell cycle entry and NA/VM. Around 40% of all cancers are associated with MAPK dysregulation [223] yet there is still a lack of precision therapies that target pathways activated by mechanical forces.

The MAPK signalling pathway shares several regulatory mechanisms with the Hippo pathway to control cell proliferation and apoptosis. Downregulation of the tumour suppressive Hippo pathway leading to YAP/TAZ activation, RUNX2 stabilisation [224] and TEAD transcription factor onset is a key target in several cancers [225, 226] including Ewing sarcoma. The potent YAP/TAZ/TEAD complex inhibitor verteporfin was shown to reduce metastatic relapse in animal models [227]. Shear stress increases MAPK1/3 activity [220–222, 228] as well as mechanosensitive YAP/TAZ [229] that together enhance cell proliferation by positively regulating the cell cycle, DNA replication, DNA repair and mitosis. A correlation between the IGF1 receptor (IGF1R) and the nuclear localisation of YAP/TAZ was identified in patient tissue samples highlighting the potential prognostic value of these markers for osteosarcoma progression [230].

Most studies investigating shear stress and metastasis simulate the continuous mechanical forces observed in vivo. Although some studies focus on the targeting of forces there is lack of knowledge on how the changes in shear stress will affect phenotypes in bone sarcoma cells. Dynamic shear stress regulates mesenchymal and hematopoietic stem cells in both an osteo-protective and an osteo-destructive manner [231–235]. Biological response to sheer stress is measured and controlled by changes in amplitude, duration and rhythmicity of the mechanical shear stress forces [233]. Differential drug responses have been observed when loading is applied [231, 233].

### The actin network in metastatic cells

The physical properties of cancer cells can influence metastatic processes. One key distinguishing feature of metastatic cells is their high deformability, which is linked to cell stiffness and can be measured by atomic force microscopy as Young’s modulus. Cell stiffness can be determined by the distribution and organisation of the actin cytoskeleton [236]. Actin is an important structural protein that occurs in all cell types and forms the scaffolding of the cell, i.e. the cytoskeleton. Genes encoding for actin are first transcribed and translated into G-actin spherical monomers. In the more stable ATP state, these actin monomers combine with the rapidly growing barbed end of the filament and form fibrous F-actin strands. Filamentous actin contributes to multiple cellular functions including migration and invasion. During malignant transformation, actin cytoskeleton dependent functions are dysregulated [237]. The cytoskeletal structure converts from a well organised network to a more irregular arrangement ultimately resulting in decreased stiffness and increased deformability. Studies investigating human osteoblast stiffness have revealed the inherent presence of large focal adhesions and actin stress fibres [238]. Conversely, osteosarcoma cells have small, sparse focal adhesions and fewer actin fibres making them ‘softer’ [238]. These findings have been independently observed in metastatic murine osteosarcoma (LM8 cells) when compared to non-metastatic cells [239].

Cytoskeletal filaments in tumour cells generate physical forces termed solid stress as cells expand and interact with the dense stromal cell-extracellular matrix of the host tissue. Cytoskeletal ‘mechanosensors’ enable cancer cells to detect increased traction forces, activating Rho GTPases to initiate signaling cascades and to reorganise the cytoskeleton [240]. Rho GTPases CDC42, RHOJ and RHOA are over expressed in multiple tumour types and act as key molecular switches between their active GTP bound form and their inactive form in combination with guanine nucleotide exchange factors promoting GDP to GTP exchange and GTPase activating proteins regulating GTP hydrolysis [241]. Enhanced CDC42 activity supports genomic stability and activates DNA damage repair pathways in hematopoietic stem cells, mesenchymal stem cells and cancer cells [242–245]. CDC42 is considered a hub gene in the osteosarcoma context [246]. In Ewing sarcoma, CDC42 negatively regulates the BAF chromatin remodelling complex and influences proliferation [247, 248]. RHOJ controls drug resistance by enhancing replicative stress response, activates DNA damage response and enables tumour cells to rapidly repair DNA lesions induced by chemotherapy [249]. In osteosarcoma, RHOA is upregulated and associated with a poor 5-year metastasis free survival rate [250]. In Ewing sarcoma, the hypoxia activated neuropeptide Y/Y5 receptor/RHOA pathway triggers chromosomal instability, bone metastasis and chemoresistance [251]. LOXHD1 can affect cytoskeletal reorganisation in response to hypoxia through HIF1 A stability [252]. HIF1 A and YBX1 activation promotes metastasis in high-risk sarcomas including bone [253–256].

Pharmacological inhibition to Rho GTPase overactivation leads to reorganisation of the actin cytoskeleton in hematopoietic and mesenchymal stem cells upon stress [242, 243]. In mouse models of colorectal cancer this therapeutic approach has shown increased anti-cancer T cell immunity [257], therefore, targeted inhibition of CDC42 and other GTPases can restructure the actin cytoskeleton and restore cellular stiffness, which holds therapeutic promise for reducing bone sarcoma migration and invasion.

### Targeting hypoxia: boosting response in bone sarcoma therapy

Hypoxia is a central component involved in tumour progression and is a target for therapeutic intervention [258–260]. Hypoxia results from abnormal angiogenesis and the heightened demand for oxygen and glucose required by the accelerated growth and mitosis of cancer cells [261, 262]. The imbalance between oxygen supply and consumption within a tumour leads to a low oxygen environment [261]. In experimental osteosarcoma models, hypoxia has been shown to induce EMT, partially mediated by HIF1 A and PDGFRB [263]. This signalling cascade promotes cytoskeletal rearrangements and mesenchymal like phenotypes essential for metastasis. Silencing PDGFRB or interfering with HIF1 A both significantly reduce metastatic propensity [263, 264]. Ongoing trials are exploring PDGF/PDGFR or HIF inhibitors combined with existing agents in solid and soft tissue sarcomas with promising results [258, 265].

Hypoxia also influences tumour metabolism and immune interactions [266]. High-risk tumours show upregulation of hypoxia and lactate metabolism related genes including *MAFF* and *COL5 A2* while protective genes such as *SQOR* and *PFKFB2* correlate with enhanced immune infiltration [266]. Hypoxia upregulates proteins such as *STC2* and *TMEM45 A*, which have been shown to drive proliferation, migration and metastasis in solid cancers [266]. Elevated STC2 levels reduce NK and T cell infiltration and increase cancer associated fibroblast (CAF) activity contributing to immune evasion and poor patient outcomes [267]. Hypoxia induced miR-18b-5p contributes towards a pro-metastatic TME by suppressing the *PHF2* tumour suppressor [268]. Elevated levels of this miRNA correlate with unfavourable clinical outcomes [268]. The use of hypoxia activated prodrugs [269] and oxygen nanogenerators [270] has been shown to induce anti-tumour immunity.

Proteomic changes in osteosarcoma under hypoxic conditions include the upregulation of collagen biosynthesis proteins P4HA1, PLOD1, PLOD2 and LOX and antioxidant enzymes including PRDX1, which also contribute to aggressive tumour behaviour and lung metastases [264]. These proteomic changes or adaptations confer osteosarcoma cell survival advantage through hypoxia driven pathways leading to invasion and therapy resistance. Emerging therapeutic strategies seek to exploit these hypoxia driven adaptations. For example, photothermal nanoenzymes [271] and capsaicin (CAP) mediated enhancements to photodynamic therapy target the hypoxia impact on tumour metabolism [272]. CAP not only reduces oxygen consumption via TRPV1 activation but also induces ferroptosis and post-transcriptionally inhibits HIF1 A counteracting hypoxia driven therapy resistance [272].

### Matricellular proteins promote metastasis

A family of secreted cysteine rich extracellular matrix proteins termed matricellular proteins have emerged as major positive/negative contributors to metastatic progression [273]. One protein family termed CCN comprises six matricellular proteins that regulate cell adhesion, migration, proliferation, survival and differentiation. CCN3 is associated with high risk for lung and/or bone metastases in both Ewing sarcoma and osteosarcoma [274, 275]. *CCN1* is located on chromosome 1 that often undergoes gain or amplification in osteosarcoma [276]. CCN1 expression is higher in osteosarcoma tumours compared to normal bone tissue and further increased in metastatic tissues [277] (Fig. 3). CCN1 promotes NA/VM and favours lung dissemination [277, 278]. Due to partial identity with the insulin like growth factor binding proteins, CCN1 influences IGF1 and IGF1R expression and IGF1R downstream signaling including JNK dependent pathways [279]. Phase I/II trials evaluating IGF1R antibodies in sarcoma have produced mixed results. Despite the small number of patients with osteosarcoma a few stable and partial/complete responses were reported [280–283].

### Cell plasticity enables metastable phenotypes

Cell plasticity is defined as the ability of a cell to actively or passively and reversibly change its phenotype [284–286]. Plasticity is a key feature in several physiological processes including stem cell maintenance, wound healing and cell reprogramming, however, if uncontrolled and aberrantly activated, it can drive cancer development. In oncology, cell plasticity plays key roles in MDR and disease progression [287].

The concept of tumour cell plasticity refers to the activation of developmental programmes that are closely correlated with EMT, cancer stem cell (CSC) acquisitional properties and transdifferentiation potential, the latter of which may arise following drug exposure [187]. Tumour cell plasticity through altering cellular differentiation programmes can lead to tissue disorganisation and promotes the creation of a tumour niche. The same factors released by the microenvironment as well as the microenvironment itself influence the plasticity of the niche cells, preferentially activating selected mechanisms over others [287]. Tumour cell plasticity comprises all the mechanisms of tumour reprogramming not related to genomic/genetic alterations including epigenetic modifications, signalling pathway alterations and environmental interactions [288].

Cell plasticity mechanisms in metastasis can be varied. EMT processes are amongst the most studied and have been well described in carcinomas. Epithelial cancer cells undergo a transition to a mesenchymal phenotype characterised by the loss of basal and apical polarity followed by the breakdown of all cell–cell contacts [289]. Less is known about EMT/MET mechanisms in tumours of mesenchymal origin, including bone, that by their inherent nature comprise stem cell like features including clonal expansion and migratory capacity and lack of apical-basal junctions [290]. Mesenchymal tumours do not apparently need to activate EMT/MET in the same vein, or timeframe, as epithelial cancers.

Expression of epithelial cell markers such as tight junction proteins ZO-1 and CLDN1 have been observed in Ewing sarcoma [291]. Though only an observation and not mechanistically investigated this discovery might indicate that MET is important for progression and dissemination even in mesenchymal tumours [287]. Interaction between TNFRSF11 A and its ligand TNFSF11 was shown to increase osteosarcoma migration, invasion and metastasis via EMT induction [292]. The TNFRSF11 A-TNFSF11 axis induced EMT by activating the NF-κB pathway, which could be reversed in vitro by dimethyl fumarate (DMF) [292]. Some sarcoma cells can exist in a transient state known as ‘partial EMT’ where both mesenchymal and epithelial states coexist in a hybrid form [293, 294]. Though the full mechanistic rationale is unclear, it is speculated that a ‘metastable’ phenotype enables circulating cells to alter their morphology and resist migration stress, leading to higher aggressiveness and metastatic potential. In sarcomas, these phenotypic traits are further evidenced by the clinical categorisation into epithelial like sarcomas, for example, Ewing sarcoma, synovial sarcoma and epithelioid sarcomas, and mesenchymal like sarcomas, for example, osteosarcoma and chondrosarcoma plus the existence of entities and subtypes with both characteristics [293, 295].

### Transcription factors and non-coding RNAs

EMT associated transcription factors including SNAI1, TWIST1 and ZEB1 [296] contribute toward metastatic bone sarcoma (Fig. 3). In osteosarcoma, TGFβ can escape miR-124 negative regulation [297] and trigger SNAI1 expression to cause epithelial marker downregulation while upregulating mesenchymal markers, inducing EMT in vivo [287, 298]. Studies have exploited the relationship between EMT/MET transcription factors and miRNAs as these can be used either as prognostic biomarkers or as therapeutic targets. For example, in osteosarcoma, SNAI1 expression is associated with miR-145 downregulation [299]. Indeed, miR-145 overexpression induced by a miR-145 agomiR resulted in a dampened ability of osteosarcoma cells to migrate and invade in vitro, attenuated SNAI1 and CDH1 expression, thus reverting EMT [299]. ZEB1 is overexpressed in osteosarcoma correlating with greater cancer cell migratory and invasive capacity [300]. ZEB1 overexpression is associated with low miR-144-3p expression [301]. Rescued miR-144-3p subsequently downregulates ZEB1 reducing osteosarcoma metastatic capacity [301]. In Ewing sarcoma, a delicate balance between EWSR1::FLI1 and miR-145 has been reported as an essential oncogenic component [302]. MiR-145 is the top EWSR1::FLI1 repressed miRNA in a positive feedback loop with the *EWSR1*::*FLI1* transcript [302]. Further, a degree of stemness is maintained by downregulated miR-145, which is known to suppress stemness transcription factors including POU5 F1, SOX2, KLF4 and MYC [302].

Transcription factor: miRNA regulation of EMT/MET occurs not only post-transcriptionally but also transcriptionally via epigenetic factors including histone de/methylation and other non-coding RNA species [303]. These regulatory mechanisms hold distinct importance in paediatric tumours where there is a lower mutational burden [304]. One example is upregulation of the histone deacetylase HDAC5 in osteosarcoma, which via its gene repressive function, activates downstream *TWIST1* expression, a known oncogene and EMT driver [305]. The *TWIST1* negative regulator, deactivated by HDAC5, was not reported, though independently, miR-22 is a known direct regulator of *TWIST1* [306] where low miR-22 levels in osteosarcoma tumours contributes to EMT and disease progression through *TWIST1* [306]. Similarly, long non-coding RNAs (lncRNAs) are both oncogenic and tumour suppressive through activating or inhibiting EMT associated transcription factors as well as the EMT/MET processes. LncRNA *AFAP1-AS1* is pathogenic in osteosarcoma [307]. *AFAP1-AS1* plays an important role in multiple cell processes including apoptosis, the cell cycle, migration and invasion. *AFAP1* knockdown induces G0 arrest, apoptosis and suppresses EMT as well as NA through the inhibition of RHOC/ROCK1/p38MAPK/TWIST1 signalling [307]. The maternally expressed paternally imprinted lncRNA *H19* is highly upregulated in osteosarcoma [308], reciprocally imprinted and regulated with its neighbouring gene *IGF2* [309]. *H19* is an embryonic morphogen and mediator of sonic hedgehog (SHH) signalling required for stem cell division [308] and has clinical significance in Beckwith–Wiedemann syndrome, which predisposes individuals to cancer development [310].

Over the past decade and particularly during the SARS-CoV-2 pandemic, major technological innovation and research investment have enabled synthetic RNA molecules to become promising therapeutic tools [311]. Only ~ 15% of human proteins are ‘druggable’ meaning RNA therapies and similar modalities are important future therapeutics. Messenger RNA (mRNA), small RNA (sRNA) [312–315] and antisense oligo (ASO) therapies similar to nusinersen used to treat spinal muscular atrophy could be designed as targeted therapies in bone sarcomas.

### RNA-binding proteins (RBPs) as post-transcriptional mRNA regulators

RBPs are essential post-transcriptional regulators [316]. RBP dysregulation significantly impacts tumour cell plasticity mechanisms associated with EMT/MET as well as cancer cell migration and invasion [317]. The RBP IGF2BP3 serves as a prognostic biomarker for patients with Ewing sarcoma where its high expression correlates with poor patient survival through increased metastasis [318]. IGF2BP3 is an oncofoetal protein synthesised de novo in cancer where it promotes drug resistance and metastasis via IGF2-dependent and IGF2-independent mechanisms through *IGF1R* RNA-binding [318]. In Ewing sarcoma, IGF2BP3 loss promotes the downregulation of *IGF1R* and a decreased biological response to IGF1 [318]. Compensatory activation of the insulin receptor (IR) and its mitogenic ligand IGF2 is triggered in some Ewing sarcoma cells in response to IGF2BP3 mediated *IGF1R* loss [318]. These findings have therapeutic implications because cells with a decreased expression of the IGF2BP3/*IGF1R* axis but an increased expression of the IR/IGF2 loop display higher sensitivity to the dual inhibitor linsitinib [318].

### Cell surface proteins as targeted therapy and immunotherapy targets

A distinctive Ewing sarcoma feature is high CD99 expression, a membrane protein involved in regulating several biological processes including cell adhesion, migration and apoptosis through the PI3 K/RAS/MAPK signalling pathways [319–322]. These actions underscore CD99 involvement in maintaining the Ewing sarcoma cell undifferentiated state. CD99 is expressed in a balance with EWSR1::FLI1, which instead appears to drive cells toward a proliferative and neuronal state [322]. The combined effects are crucial for shaping the Ewing sarcoma phenotype. CD99 knockdown in human Ewing sarcoma cell lines reduced their ability to form tumours and bone metastases when xenografted into immunodeficient mice [322]. CD99 knockdown caused neurite outgrowth, increased beta-III tubulin expression and neural differentiation [322]. CD99 has been the target of several new therapies [323] including modulated clofarabine [324] and could be the focus of emerging immunotherapies.

A more recent surfaceome analyses revealed many new Ewing sarcoma cell surface targets including ENPP1 and CDH11 in addition to known IL1RAP, STEAP1, ADGRG2 and CD99, providing newer cell surface targets for immunotherapeutic application in Ewing sarcoma [325]. In metastatic osteosarcoma, the purinergic receptor P2RX7 B isoform is expressed and clinically actionable via the A740003 agonist [326]. BT1769, an osteosarcoma cell surface MMP14 targeted bicycle toxin conjugate, demonstrated anti-tumour activity, high target affinity and a favourable pharmacokinetic profile in patient derived xenograft models [327]. The GD2 ganglioside [328] and ALPL [329] are clinically actionable through CAR T cells.

### Precursors to metastasis: circulating tumour cells (CTCs)

Circulating tumour cells (CTCs) are the ultimate products of local intravasation and are thought to be the physical effectors of metastasis (Figs. 1 and 3). Investigating the ‘seeds’ of disease spread might reveal the key phenotypes including genetic, biological and mechanical that impact their likelihood to form metastases. The earliest contemporary CTC studies were performed in prostate cancer models, which showed that human tumours transplanted into nude mice only delivered viable “*circulating metastatic cells*” to the blood if they were placed in the orthotopic tissue. Tumours transplanted into ectopic sites did not lead to metastasis [162] suggesting a biological importance for the primary tissue site in metastatic CTC generation. Since this work, most studies have focused on CTC isolation and prognostic quantification [330–333] meaning there remains multiple unanswered questions on their basic biology. Pertinent questions include: (i.) How are CTCs are generated in the first place? Conflicting observations even in the same cancer type show CTCs can be generated by intratumour hypoxia [334], conversely, low density normoxic lesions display more stemness and produce more CTCs [335]. (ii.) From where within the tumour do CTCs depart? (iii.) Are CTCs Darwinian selected clones competent of dissociation, migration and colonisation or are they stressed cells forced to intravasate because their environment in the primary site has become unfavourable? (iv.) More modestly, but by no means less dangerously, are CTCs passively shed tumour cells participating in a highly ineffective process where one or two out of tens of thousands might eventually achieve metastasis? Addressing any of these propositions requires the consideration of nutrient availability [336], new metastatic driver genes [337, 338] and microenvironmental stimuli [334, 339] that might influence the systemic phenotype [340]. Even the innocuous timing of these events, for example, circadian rhythm, might be clinically relevant [341, 342].

One of the first studies investigating CTC mechanics in osteosarcoma used the GFP-HOS/MNNG human cell line implanted into nude mice and collected blood samples before and after tumour formation when exposed to ifosfamide [343]. CTC quantification via flow cytometry and DEPArray showed that parallelly to increased tumour growth, CTC number increased in a time dependent manner and were detectable prior to any palpable tumour mass or lung metastases [343]. Ifosfamide reduced tumour volume and metastatic foci, but the number of CTCs increased [343]. Two independent CTC derived cell lines were developed ex vivo and compared with the parental tumour cells. Proliferation, migration and invasion were not significantly different between parental HOS/MNNG and the HOS/MNNG derived CTCs [343]. Measuring the expression of four genes associated with metastasis, *CD99*, *ADAM8*, *ENDRA* and *LTK*, showed that *CD99* expression had been achieved in the CTC lines but this gain was inadequate in forming lung metastases when exposed to ifosfamide [343]. The paradoxical effects of ifosfamide on reducing tumour volume but increasing CTC number could be explained by the vasculature network in bone tumours causing a higher ifosfamide bioavailability and tumour cell release into the circulation. After implant, HOS/MNNG cells establish cell contact with their microenvironment, gradually proliferate and establish an osteoid matrix [186, 205]. The fragile interface between tumour cells and the tissue microenvironment at an early disease stage could explain tumour cell, but not ‘truly metastatic’ cell, release after ifosfamide exposure. The physical and molecular properties such as deformability and new driver mutations, respectively, enabling truly metastatic CTC extravasation and the formation of metastases at distant sites might not be acquired until a later stage in a time dependent and possibly selective manner [343].

Transcriptome wide reprogramming is a metastatic hallmark. Detecting the underlying master regulators that drive pathological gene expression in cancer cells is a challenge. Our own work in osteosarcoma used an integrated analytical approach combining whole tumour RNA-seq and single CTC scRNA-seq of patient samples, cell lines and animal models to search for metastatic master regulators [54]. A co-expression network was built on all genes observed to be expressed in osteosarcoma derived CTCs through deep sequencing. We searched for gene modules, described as a set of co-expressed genes to which the same set of transcription factors binds, that were enriched for differentially expressed genes (DEGs). Gene modules, 26 in total, enriched for DEGs were used to reveal metastasis associated gene expression in CTCs [54]. Using the patient derived CTC datasets as a guide, we generated a xenograft mouse model to mechanistically reveal a tumour-immune cell interaction that drives CTC production and lung metastasis [54]. In patient derived CTCs, there was an abundance of mitochondrial gene expression including cytochrome c oxidase I, II and III, NADH: ubiquinone oxidoreductase core subunits 1–4 and cytochrome b [54]. These RNAs and subsequent proteins are central to OXPHOS and describe a shift in CTC intracellular metabolic profile from glycolysis, which is more ubiquitous in tumour cells [344]. Also detectable in CTC transcriptomes at single-cell resolution there was evidence of circulatory stress tolerance with *HBB* and ubiquitin C expression [54]. There were stem cell and developmental skeletogenesis gene markers including *MET*, *FGF10*, *FN1*, *TGFB2* and miR-140 driven *RUNX2* expression [54, 345] (Fig. 3). For the latter gene, we developed the small molecule compound CADD522, which significantly increased metastasis free survival in Ewing sarcoma and osteosarcoma animal models. CADD522 is undergoing dedicated toxicology studies [256].

*MMP9* was also highly expressed in osteosarcoma derived CTCs [54]. In a concerted approach to avoid the challenges experienced in previous studies, i.e. MMP9 inhibitor drugs have had limited success in patient trials [346], we opted to target the *MMP9* upstream transcriptional regulator MAPK7. We cloned highly metastatic human osteosarcoma 143B cells with stably expressed short hairpin RNA (shRNA) to suppress *MAPK7* expression. Mutant cells showed a significantly reduced ability to colonise the lungs [54]. We used in vivo fluorescence imaging to show that active MMP9 laterally increased with tumour growth in controls. Tumour cells harbouring shMAPK7 showed significantly reduced fluorescence signal in both primary and metastatic lesions. MMP9 signal was mostly localised to the tumour edge, that is, the invasive margin [54]. MAPK7 and therefore MMP9 silencing significantly minimised M2 like TAM infiltration at the tumour site, M1 to M2 polarisation and lung colonisation [54]. Taken together, these results suggested that specific CTC sub-populations and their spatial interactions with TAMs are an essential step in the multifactorial cascade of bone sarcoma metastasis (Fig. 3).

### The pre-metastatic niche (PMN)

The PMN is essential for receiving CTCs and achieving systemic disease [347]. Stephen Paget first proposed the ‘seed and soil’ theory over a century ago [348]. This model proposes that disseminated cancer cells (‘seeds’) can only colonise a secondary organ (‘soil’) if the new environment is receptive and supportive for growth. Metastatic competence is therefore determined by the intricate interactions between cancer cells and the distant environment/s that they encounter. It is now well established that organs of future metastases are not simply passive receivers of CTCs but are selectively and actively modified by the tumour before metastatic spread has occurred [347]. Building on the ‘seed and soil’ analogy, tumours secrete factors that prepare (‘fertilise’) the target organ (Fig. 3). CTCs that arrest in non-PMN sites lack a supportive fertile environment and fail to colonise [347].

The lungs, bone marrow and/or other skeletal sites are the predominant foci for advanced disease in paediatric bone sarcomas [349, 350]. Primary tumour cells promote PMN formation by boosting inflammatory cytokine and chemokine release in target lung epithelial and endothelial cells and fibroblasts (Fig. 3). Cytokine release increases bone marrow derived myeloid cell recruitment whilst altering extracellular matrix composition [351]. Activation of tumorigenic signalling pathways including the CXCL12-CXCR4 axis promote EMT leading to CSC and/or cancer progenitor cells mobilising to the PMN [352]. In osteosarcoma, epigenetically downregulated *CXCL12* via DNA methyltransferase 1 (DNMT1) impairs CD8 + killer T cell homing to the tumour sites, consequently, metastatic cells evade immune mediated cytotoxicity [353]. Untreated osteosarcomas showed a positive correlation between CXCL12 concentration and the number of intratumoral lymphocytes [353]. Targeting DNMT1 in immunocompetent mouse models significantly elevated CXCL12 expression in tumours, resulting in a robust immune response and the eradication of early lung metastases [353]. Epigenetic and other therapies targeting CXCL12 have the potential for therapeutic intervention in osteosarcoma [353] leading to more favourable outcomes [354].

### Extracellular vesicles (EVs)

EVs are the ideal specialised vehicles for transmitting signals from the host tumour to shape and configure distant tissue microenvironments [355–358]. EVs are nanosized membrane bound particles that carry bioactive molecules, for example, proteins, lipids, mRNAs, miRNAs and lncRNAs [359] between cells and tissues for hetero- and homotypic intercellular communication at both paracrine and systemic levels. EVs can directly affect biological processes including NA/VM, tissue remodelling, immune and inflammatory responses and can themselves be used as biomarkers [360] (Table 2). EVs and their cargo might also affect drug response.

**Table 2:** EV related biomarkers

EV biomarker	Relevance
Oncogenic miRNA	miR-25-3p and miR-195-3p are enriched in exosomes derived from osteosarcoma. High expression correlates with metastasis. Promotes capillary and venule formation by inhibiting DKK3, cell proliferation and invasion [361, 362]. miR-487a facilitates lung metastasis through a communication mechanism between osteosarcoma cells and M2 macrophages [363]
Cancer related gene fusions and alternative splicing events	*PARAH1B2*::*FOXR1* fusion RNA transcripts in EVs derived from osteosarcoma are associated with poorer survival [364]
EV associated RNA/DNA sequences	EVs that contain repetitive element DNA sequences, notably *HSATI*, *HSATII*, *LINE1-P1*, could serve as metastasis and prognosis markers [365]
EV protein marker overexpression	CCNE1, LDHA, RB1 and COL6 A3 could serve as potential prognostic markers. PREX1, GLS, FOSL1 could serve as metastasis markers [366]. EV-packed S100 A11 stimulates an immunosuppressive PMN and tumour cell colonisation [367]. Abnormal CD63, vimentin and EPCAM expression is correlated with tumour progression [368]

Within paediatric bone sarcomas, PMN studies have mainly focussed on pulmonary metastases in osteosarcoma. Systemically administering a 143B osteosarcoma cell secretome into non-tumour bearing mice damages alveolar structure, causes inflammatory cell infiltration and produces signs of extracellular matrix remodelling and fibrosis similar to mice bearing 143B tumours [369]. In the non-tumour bearing mice there is increased fibronectin and reticulin expression as well as neutrophil infiltration, all features consistent with pulmonary PMN formation as similarly observed in tumour bearing mice [369]. EVs from 143B and SAOS2 osteosarcoma cells ‘educate’ murine lungs by inducing CD11b + myeloid cell accumulation [370] and pro-inflammatory IL6 production by mesenchymal stem cells through the selective incorporation of a membrane associated form of TGFβ [371]. Tocilizumab intravenous administration blocked IL6 and abrogated mesenchymal stem cell tumour promoting effects in vivo [371].

In vivo imaging studies using fluorescently labelled EVs from highly metastatic osteosarcoma clones have shown to preferentially target the lungs [372]. Lung specific metastatic tropism of osteosarcoma is attributed to the alteration in the immune cell populations caused by osteosarcoma derived EVs. The lung PMN in osteosarcoma is characterised by increased infiltration of granulocytic myeloid derived suppressor cells (gMDSC) that generate an immunosuppressive environment and facilitate metastatic cell colonisation. S100 A11 that is packaged into osteosarcoma EVs can activate lung interstitial macrophages that in turn initiate the influx of gMDSCs via the CXCL2-CXCR2 chemokine axis [367]. Loss of the IRF5 transcription factor in osteosarcoma promotes establishment of a lung PMN via altering the composition and trafficking of tumour derived EVs [373].

Beyond immune modulation, osteosarcoma derived EVs can contribute to metastasis by impacting NA/VM and stromal cell reprogramming. EVs from osteosarcoma cells, particularly under acidic conditions, harbour high levels of pro-angiogenic proteins and miRNAs including VEGF and miR-21-5p that enhance tumour NA and facilitate nutrient supply to metastatic lesions [374, 375] (Fig. 3). EVs carrying TGFβ can induce fibroblast-to-myofibroblast differentiation in the lungs, a process linked to increased fibroblast invasiveness and extracellular matrix remodelling, aiding metastatic cell survival and expansion [376]. Similarly, osteosarcoma secreted ANGPTL2 promotes the formation of a pulmonary PMN by recruiting neutrophils and disrupting endothelial junctions, which facilitates tumour cell extravasation and metastatic progression [377].

Ewing sarcoma derived EVs contain the *EWSR1*::*FLI1* transcript [378] and induce pro-inflammatory cytokine release from myeloid cells and direct them towards immunosuppressive phenotypes in vitro [379]. While these Ewing sarcoma EV data are consistent with classical PMN characteristics, it remains unclear if these findings translate in vivo.

Most studies to date have focussed on tumour derived EVs from homogenous culture conditions and discount the role of the TME and tumour cell heterogeneity and the resultant EV subpopulations on PMN formation. These are critical challenges that will need to be overcome to disrupt the EV mediated alterations of the target metastatic organs effectively and specifically and thereby delay metastatic progression.

### A sticky situation: aberrant wound healing programmes mediate distant colonisation

Upon arrival at the fibrotic, immunosuppressive and ‘sticky’ PMN after hemodynamic forces permit the arrest, adhesion and extravasation of cancer cells [380], CTCs (singly, in CTC clusters [381] or within CTC-macrophage complexes [382]) face significant hostile differences in their microenvironment compared with that of their original tumour. There is some consensus that a temporary exit from the cell cycle and the induction of dormancy/quiescence [383, 384] might be beneficial for survival [385], which goes some way to explain the recurrent clinical observation of relapse 1–2 years after treatment is concluded [386]. While contrary to the normally proliferative behaviour of cancer cells, one scenario is that dormancy is extrinsically imposed, for example, by the scarcity of mitogenic stimuli in the new environment and the activities of tissue resident anti-tumour immune cells [387]. Dormancy therefore allows disseminated cells and micrometastases to persist undetected and to resist therapy until reactivation occurs through mechanisms that are still unknown [387].

Upon reactivation, disseminated osteosarcoma cells induce acute alveolar epithelial injury [388]. The surrounding lung stroma adopts a chronic, non-resolving wound healing phenotype similar to other diseases associated with lung injury, for example, idiopathic pulmonary fibrosis. Metastases affected lungs display marked fibrosis deposits due to the accumulation of pathogenic, pro-fibrotic, partially differentiated epithelial intermediates and macrophages [388]. The evolutionary cascade for disseminated cancer cells at this point is to colonise as widely and as rapidly as possible and eventually achieve further metastasis-to-metastasis dissemination [389, 390] (Fig. 3).

Better understanding of the biological topography of dormancy, reactivation and adaptation to new tissue sites is likely to enable improved adjuvant approaches in the clinic [340]. Targeting tumour deposition of fibronectin in the lungs through the anti-fibrotic TKI nintedanib disrupts metastatic progression in bone sarcoma animal models [388].

## Conclusion

While bone formation starts early in fetal life [345], bone growth and remodelling continues after birth, making it one of the few organs that develops postnatally. Although disease incidence peaks in the second and third decades, bone sarcomas such as Ewing sarcoma and osteosarcoma are considered “paediatric” cancers [391]. Bone sarcomas are better considered a systemic disease with radiologically undetectable micrometastases already present at diagnosis [89]. Metastatic spread is a highly complex multistep cascade of evolutionary events performed with exquisite, but fatal, consistency across patients. Drug resistant and inoperable metastases remain the leading cause of cancer patient death. Prevention and/or treatment of the systemic disease component remains the major clinical oncology challenge.

Although the key driver mutations and several recurrent alterations have been reported in Ewing sarcoma and osteosarcoma, fragmented data from multiple small series [51] has hampered global fundamental understanding of bone sarcoma metastasis biology and, therefore, targeted therapy development. There has also historically been a limited commercial incentive for developing novel therapies for paediatric bone sarcomas [51]. The European Medicines Agency (EMA) strengthened the statutory requirement for the pharmaceutical industry to investigate new therapies in children where there is a relevant mechanism of action before marketing authorisation is granted for adults [392]. Similarly, US Congress approval of the Research to Accelerate Cures and Equity for Children (RACE) Act gave the Food and Drug Administration (FDA) powers to mandate paediatric trials for new oncology drugs developed in adult cancers with a molecular target relevant to childhood cancers.

In this panoramic overview, we have consolidated and considered the multitude of metastatic bone sarcoma mechanisms. Clinically relevant opportunities identified (Table 3) consistent with EMA and FDA legislation should increase patient trial recruitment [393, 394]. Meanwhile, EEC and FOSTER will continue to collaborate, share data, methods, samples and disseminate good practice to address key scientific questions and perform dedicated human clinical trials including those recruiting now or opening soon: rEECur, INTER-EWING-1, iEuroEwing and FOSTER-CabOs.

**Table 3:** Clinically relevant opportunities and clinically actionable targets to tackle metastatic bone sarcoma according to the reported biology

Metastatic target/s	Clinical opportunity	Bone sarcoma	Study/trial reporting the biology
**Specific gene/protein targets**			
ALPL GD2	**Immunotherapy:** CAR T cells	Osteosarcoma	[328, 329]
AXL FLT3 MET	**TKI:** cabozantinib	Ewing sarcoma, Osteosarcoma	[102] #NCT05691478 FOSTER-CABOS
BRCA RB1	**Poly-ADP-Polymerase1,2 inhibitor (PARPi):** olaparib	Osteosarcoma	[48, 395]
CD99	**Chemotherapy:** modified clofarabine	Ewing sarcoma	[324]
CDK4/6	**CDK inhibitors:** palbociclib, ribociclib, abemaciclib	Ewing sarcoma, Osteosarcoma	[396]
EWSR1::FLI1	**AgomiR:** miR-145	Ewing sarcoma	[302]
EWSR1::FLI1	**Chemotherapy:** Trabectedin	Ewing sarcoma	[397]
FGFR1-4 KIT VEGFR1-3	**TKI:** lenvatinib	Ewing sarcoma, osteosarcoma	[101, 398] rEECur, #2014–000259-99
IGFR IR	**Small molecule:** linsitinib	Ewing sarcoma	[318]
IL6	**Antibody:** tocilizumab	Osteosarcoma	[371]
MAPK7 MMP9	**Proteolysis targeting chimeras (PROTACs):** under development	Osteosarcoma	[54]
MMP14 (MT1-MMP)	**Bicyclic toxin:** BT1769	Osteosarcoma	[327]
PDGF PDGFR	**TKIs:** lenvatinib, imatinib, dasatinib, nilotinib, sorafenib, sunitinib, pazopanib	Osteosarcoma	[258, 399]
RET	**TKIs:** cabozantinib, lenvatinib, sunitinib, alectinib	Osteosarcoma	[48, 400]
RUNX2	**Small molecule:** CADD522	Ewing sarcoma, osteosarcoma	[256]
TWIST1	**AgomiR:** miR-22	Osteosarcoma	[306]
VEGFR2	**TKI:** regorafenib, cabozantinib	Ewing sarcoma	[401] INTER-EWING-1, #2021–005061-41
**Signalling pathways/cell targets**			
AMPK; mitochondrial targets	**Small molecule:** metformin	High-risk sarcomas including osteosarcoma	[402] Metform-Bone, #NCT04758000
Hypoxia	**Photothermal nanoenzymes and ferroptosis activators:** ruthenium, capsaicin	Osteosarcoma	[271, 272]
Immunometabolism	**Metabolic modifier:** Succinic acid	Melanoma, but could have use in bone sarcomas	[211]
Mitochondrial fatty acid oxidation	**FAO inhibitor:** etomoxir	All cancers	[212]
NF-κB	**Organic compound:** dimethyl fumarate	Osteosarcoma	[292]
Purinergic signalling	**P2X inhibitor:** A740003	Osteosarcoma	[326]
Rho GTPases	**Guanine nucleotide exchange factors:** NSC23766, EHop-016, MBQ-167, AZA1, AZA197, ZINC69391, 1 A-116, ITX3, CASIN, ZCL278	Sarcomas	[241]
TNFRSF11 A-TNFSF11 axis	**Antibody:** denosumab	Osteosarcoma	[403] #2021–002366-41
Tumour microenvironment/immune system	**Immunotherapies:** humanised antibodies, CAR T cell, tumour cell vaccines	Ewing sarcoma, osteosarcoma	[186]
YAP TAZ TEAD	**Photosensitiser:** verteporfin	Ewing sarcoma	[224, 227]
**Pulmonary metastases**			
Metastatic fibrosis	**TKI:** nintedanib	Osteosarcoma	[388]
Pulmonary metastases	**Repeated metastasectomies: v**ideo assisted thoracoscopic surgery (VATS)	Ewing sarcoma, osteosarcoma	[51, 78, 79]
**Systemic disease as a whole**			
Metastasis	**Immunostimulatory agent:** mifamurtide	Osteosarcoma	[404] SARCOME13, #NCT03643133
Metastatic relapse	**Cytotoxic chemotherapies:** vinorelbine, cyclophosphamide	Ewing sarcoma	iEuroEwing, #2019–004153-93
Multidrug resistance	**Drug carriers, biomaterials:** Selenium doped calcium phosphate (Se-CaP) biominerals, gallium doped bioactive glasses, Mg/Zn or Cu/Zn scaffolds, selenium oxide, iron oxide nanoparticles	Osteosarcoma	[168, 169, 175–177]
Post-operative metastatic relapse	**Perioperative therapies:** propofol instead of desflurane, analgesics, immuno-nutrition, beta blockers, anti-inflammatories	Ewing sarcoma, Osteosarcoma	[113]

## Data Availability

No datasets were generated or analysed during the current study.

## Abbreviations

ABCB1: ATP binding cassette subfamily B member 1
ABCC1: ATP binding cassette subfamily C member 1
ADAM8: ADAM metallopeptidase domain 8
ADGRG2: Adhesion G protein-coupled receptor G2
ALP: Alkaline phosphatase
ALPL: Alkaline phosphatase (gene)
ANGPTL2: Angiopoietin like 2
ASO: Antisense oligonucleotide
ATM: ATM serine/threonine kinase
ATRX: ATRX chromatin remodeler
BAP1: BRCA1 associated protein 1
BRCA2: BRCA2 DNA repair associated
C1QC: Complement C1q C chain
CADD522: Computer aided drug design 522
CAF: Cancer associated fibroblasts
CAP: Capsaicin
CAR: Chimeric antigen receptor
CCL2: C–C motif ligand 2
CCL18: C–C motif chemokine ligand 18
CCN1: Cellular communication network factor 1
CCNE1: Cyclin E1
CD45RO: Cluster of differentiation 45 RO isoform
CD63: Cluster of differentiation 63
CD99: Cluster of differentiation 99
CDC42: Cell division cycle 42
CDH1: Cadherin 1
CDH11: Cadherin 11
CDK: Cyclin dependent kinase
COL1 A1: Collagen type I alpha 1 chain
COL5 A2: Collagen type V alpha 2
COL6 A3: Collagen type VI alpha 3 chain
CRP: C reactive protein
CSC: Cancer stem cell
CT: Computerised tomography
CTC: Circulating tumour cell
Cu/Zn: Copper, zinc
CXCL2: C-X-C motif chemokine ligand 2
CXCR2: C-X-C motif chemokine receptor 2
DEG: Differentially expressed gene
DMF: Dimethyl fumarate
DIO3: Iodothyronine deiodinase
DIO3OS: DIO3 opposite strand upstream RNA
DKK3: Dickkopf WNT signaling pathway inhibitor 3
DLK1: Delta like non-canonical Notch ligand 1
EEC: EURO EWING Consortium
EMA: European Medicines Agency
EMT: Epithelial-to-mesenchymal transition
ENDRA: Endothelin receptor type A
ENPP1: Ectonucleotide pyrophosphatase/phosphodiesterase 1
EPCAM: Epithelial cell adhesion molecule
ETV6::RUNX1: ETS variant transcription factor 6 fused to RUNX family transcription factor 1
EV: Extracellular vesicle
FANCA: FA complementation group A
FAO: Fatty acid oxidation
FDA: Food and Drug Administration
FET::ETS: FET protein family fused to ETS transcription factor family
FGF10: Fibroblast growth factor 10
FN1: Fibronectin 1
FOSL1: AP-1 transcription factor subunit
FOSTER: Fight osteosarcoma through European research
GDP: Guanosine diphosphate
GFP: Green fluorescent protein
GLS: Glutaminase
gMDSC: Granulocytic myeloid derived suppressor cells
GTP: Guanosine triphosphate
HBB: Hemoglobin subunit beta
HDAC5: Histone deacetylase 5
HIF1 A: Hypoxia-inducible factor 1-alpha
HSATI: Human satellite 1
HSATII: Human satellite 2
IDH: Isocitrate dehydrogenase
IGF1/2: Insulin like growth factor ½
IGF1R: Insulin like growth factor 1 receptor
IGF2BP3: Insulin-like growth factor 2 mRNA-binding protein 3
IL6: Interleukin 6
IL10: Interleukin 10
IL1RAP: Interleukin-1 receptor accessory protein
IR: Insulin receptor
IRF5: Interferon regulatory factor 5
JNK: C-Jun N-terminal kinases
KLF4: KLF transcription factor 4
LDH: Lactate dehydrogenase
LDHA: Lactate dehydrogenase A
LINE1-P1: Long interspersed nuclear element-1
lncRNAs: Long non-coding RNAs
LOX: Lysyl oxidase
LOXHD1: Lipoxygenase homology PLAT domains 1
LTK: Leukocyte receptor tyrosine kinase
MAFF: MAF bZIP transcription factor F
MAPK7: Mitogen-activated protein kinase 7
M-CSF: Macrophage colony-stimulating factor
MDC1: Mediator of DNA damage checkpoint 1
MDR: Multidrug resistance
MEG3/8: Maternally expressed 3/8
MET: MET proto-oncogene
MET: Mesenchymal-to-epithelial transition
Mg/Zn: Magnesium zinc
MMP9: Matrix metallopeptidase 9
MMP14: Matrix metallopeptidase 14
mRNA: Messenger RNA
MUTYH: MutY DNA glycosylase
MYC: MYC proto-oncogene, bHLH transcription factor
NA: Neoangiogenesis
NF-κB: Nuclear factor kappa-light-chain-enhancer of activated B cells
NK: Natural killer
NLR: Neutrophil–lymphocyte ratio
NOTCH: Neurogenic locus notch homolog protein
NUMA1: Nuclear mitotic apparatus protein 1
OC: Osteocalcin
OXPHOS: Oxidative phosphorylation
P2RX7: Purinergic receptor P2X 7
p38MAPK: P38 mitogen-activated protein kinases
P4HA1: Prolyl 4-hydroxylase subunit alpha 1
ARAH1B2::FOXR1: ARIH2 ariadne RBR E3 ubiquitin protein ligase 2 fused to Forkhead box protein R1
PDGF: Platelet-derived growth factor
PDGFR: Platelet-derived growth factor receptor
PDGFRB: Platelet-derived growth factor receptor beta
PFKFB2: PFK-2/FBPase-2, 6-phosphofructo-2-kinase/fructose-2,6-biphosphatase 2
PHF2: PHD finger protein 2
PLOD1: Procollagen lysyl hydroxylase 1
PLOD2: Procollagen lysyl hydroxylase 2
PLR: Platelet-to-leukocyte ratio
PMN: Pre-metastatic niche
POU5 F1: POU class 5 homeobox 1
PRDX1: Peroxiredoxin 1
PREX1: Phosphatidylinositol-3,4,5-trisphosphate dependent Rac exchange factor 1
PROTAC: Proteolysis targeting chimera
PTEN: Phosphatase and tensin homolog
RACE: Research to Accelerate Cures and Equity (RACE) for Children Act
RB1: RB transcriptional corepressor 1
RBP: RNA-binding protein
RECQL4: RecQ like helicase 4
RET: RET proto-oncogene
RHOA: Ras homolog family member A
RHOC: Ras homolog family member C
RHOJ: Ras homolog family member J
RNAi: RNA interference
ROCK1: Rho associated coiled-coil containing protein kinase 1
RTL1: Retrotransposon Gag like 1
RUNX2: RUNX family transcription factor 2
S100 A11: S100 calcium binding protein A11
scRNA-seq: Single-cell RNA sequencing
Se-CaP: Selenium doped calcium phosphate
shMAPK7: Short hairpin RNA targeting MAPK7
SNAI: Snail family transcriptional repressor
SOX2: SRY-box transcription factor 2
SPP1: Osteopontin
SQOR: sulphide: Quinone reductase
SQSTM1: Sequestosome 1
sRNA: Small RNA
STAG2: Cohesin subunit SA-2
STAT3: Signal transducer and activator of transcription 3
STC2: Stanniocalcin 2
STEAP1: STEAP family member 1
TAM: Tumour associated macrophage
TAZ: Transcriptional co-activator with PDZ-binding motif
TCF7L1: Transcription factor 7 like 1
TGFB2: Transforming growth factor beta-2
TGFβ: Transforming growth factor beta
TKI: Tyrosine kinase inhibitor
TME: Tumour microenvironment
TMEM45 A: Transmembrane epididymal protein 1
TNFSF11: TNF superfamily member 11
TNFRSF11 A: TNF receptor superfamily member 11a
TP53: Tumour protein p53
TRAP: TNF receptor associated protein 1
TREM2: Triggering receptor expressed on myeloid cells 2
TRPV1: Transient receptor potential cation channel subfamily V member 1
TWIST1: Twist family bHLH transcription factor 1
VATS: Video assisted thoracoscopic surgery
VEGF: Vascular endothelial growth factor
VM: Vasculogenic mimicry
WNT: Wnt signaling pathway
WRN: WRN RecQ like helicase
YAP1: Yes1 associated transcriptional regulator
YBX1: Y-box binding protein 1
ZEB1: Zinc finger E-box-binding homeobox 1
